# A change for the antibacterial treatment policy to decrease carbapenem consumption at a hematopoieteic stem cell transplant center

**DOI:** 10.1186/2047-2994-4-S1-P175

**Published:** 2015-06-16

**Authors:** G Metan, L Kaynar, N Yozgat, F Elmali, C Altay Kurkcuoglu, E Alp, M Cetin

**Affiliations:** 1 Infectious Diseases and Clinical Microbiology, Erciyes University Faculty of Medicine, Kayseri, Turkey; 2 Infectious Diseases and Clinical Microbiology, Hacettepe University Faculty of Medicine, Ankara, Turkey; 3 Hematology, Erciyes University Faculty of Medicine, Kayseri, Turkey; 4 Hospital Pharmacy Unit, Erciyes University Hospital, Kayseri, Turkey; 5 Biostatisitics, Erciyes University Faculty of Medicine, Kayseri, Turkey; 6 Infection Control Committee, Erciyes University Hospital, Kayseri, Turkey

## Introduction

Carbapenems are widely used in the treatment of febrile neutropenia. However, this resulted a high rate of carbapenem resistance in our hematopoieteic stem cell transplantation (HSCT) center.

## Objectives

Here, we want share the results of antibacterial usage policy which allowed to decrease the consumption of carbapenems.

## Methods

An interventation in two stages was introduced in HSCT center. At the first eight months of 2014, carbapenems remained to be the first choice for febrile neutropenic patients while the use of piperacillin/tazobactam (TZP) was encouraged in patients with stable clinical condition. When blood cultures were reported as negative and patient was clinically stable the carbapenem/TZP treatment was stopped regardless of continious fever and neutrophil count. From October 2014, TZP (with prolonged infusion) with or without amikacin replaced carbapenems as the first line therapy of neutropenic fever. Daily defined dosages (DDD) per 1000 patient days were calculated for all antibiotics by the hospital pharmacist for each year.

## Results

A total of 913 admissions with 11,544 patient days were followed in 2013; and 1,072 admissions with 11,843 patients days were followed in 2014. An increase was observed in the rate of nosocomial pneumonia, central line associated bacteraemia and as well as the rate of ESBL and carbapenem resistance in gram negative bacilli infections in 2014 when compared with 2013.

The DDDs/1000 patient days for imipenem, meropenem, vancomycin, teicoplanin, daptomycin, linezolid, colistin, TZP and amikacin in 2013 and 2014 were as follows; 201 *vs* 19; 1,578 *vs*1,092; 533 *vs* 251; 205 *vs* 159; 56 *vs* 14; 76 *vs* 26; 188 *vs* 154; 157 *vs* 254; and amikacin 5 *vs* 41.

## Conclusion

Despite the rates of nosocomial infections and antibiotic resistance increased relatively, we were able to decrease the consumption of not only carbapenems but also glycopeptides. The sustainability of such intervention needs to be monitored continuously.

## Disclosure of interest

G. Metan Grant/Research support from: Associates of Cape Cod, Conflict with: Member of Advisory board for Pifizer, Gilead, Astellas, L. Kaynar: None declared, N. Yozgat: None declared, F. Elmali: None declared, C. Altay Kurkcuoglu: None declared, E. Alp: None declared, M. Cetin: None declared.

